# Global Screening and Functional Identification of Major HSPs Involved in PVY Infection in Potato

**DOI:** 10.3390/genes13040566

**Published:** 2022-03-23

**Authors:** Kun Li, Ruhao Chen, Zheng Tu, Xianzhou Nie, Botao Song, Changzheng He, Conghua Xie, Bihua Nie

**Affiliations:** 1 Key Laboratory of Potato Biology and Biotechnology (HZAU), Ministry of Agriculture and Rural Affairs, Huazhong Agricultural University, Wuhan 430070, China; likun1008@webmail.hzau.edu.cn (K.L.); wzdhhhy@126.com (R.C.); tuzheng@webmail.hzau.edu.cn (Z.T.); songbotao@mail.hzau.edu.cn (B.S.); xiech@mail.hzau.edu.cn (C.X.); 2 Key Laboratory of Horticulture Plant Biology (HZAU), Ministry of Education, Huazhong Agricultural University, Wuhan 430070, China; 3 Hunan Provincial Key Laboratory of Crop Germplasm Innovation and Utilization, Hunan Provincial Engineering Research Center for Potatoes, Hunan Agricultural University, Changsha 410128, China; hecz@hotmail.com; 4 Fredericton Research and Development Centre, Agriculture and Agri-Food Canada, 850 Lincoln Road, Fredericton, NB E3B 4Z7, Canada; xianzhou.nie@agr.gc.ca

**Keywords:** potato, PVY, HSP40 (DnaJ), HSP70, HSP90

## Abstract

HSP40 (also known as DnaJ), HSP70, and HSP90 are major heat shock protein (HSP) families that play critical roles in plant growth and development and stress adaption. Recently, several members of the three HSP families were reported to be widely involved in the plant host-virus interactions. However, their global expression profiles and core members recruited by viruses are largely unknown. In this study, a total of 89 *StDnaJs* were identified from a genome-wide survey, and their classification, phylogenetic relationships, chromosomal locations, and gene duplication events were further analyzed. Together with 20 *StHSP70s* and 7 *StHSP90s* previously identified in the potato genome, the global expression patterns of the members in 3 HSP families were investigated in 2 potato cultivars during *Potato virus Y* (PVY) infection using RNA-seq data. Of them, 16 genes (including 8 *StDnaJs*, 6 *StHSP70s*, and 2 *StHSP90s*) were significantly up- or downregulated. Further analysis using qRT-PCR demonstrated that 7 of the 16 genes (*StDnaJ06*, *StDnaJ17*, *StDnaJ21*, *StDnaJ63*, *StHSP70-6*, *StHSP70-19*, and *StHSP90.5*) were remarkably upregulated in the potato cultivar ‘Eshu 3’ after PVY infection, implying their potential roles in the potato-PVY compatible interaction. Subsequent virus-induced gene silencing (VIGS) assays showed that silencing of the homologous genes of *StDnaJ17*, *StDnaJ21*, *StHSP70-6*, and *StHSP90.5* in *Nicotiana. benthamiana* plants dramatically reduced the accumulation of PVY, which indicated the four genes may function as susceptibility factors in PVY infection. This study provides candidate genes for exploring the mechanism of potato-PVY compatible interaction and benefits breeding work aiming to produce new cultivars with the ability to grow healthily under PVY infection.

## 1. Introduction

Potato (*Solanum tuberosum* L.) is the third-largest food crop after rice and wheat in terms of human consumption. It has become a significant strategic crop for poverty eradication, health promotion, and food security in developing countries [1]. The potato crop is constantly exposed to various abiotic and biotic stresses, such as hot, cold, and salinity, and pathogen attacks [2,3]. *Potato virus Y* (PVY), one of the top 10 plant viruses recognized in the world, is considered to be the most harmful virus affecting potatoes [2]. It was reported that PVY could cause up to a 45% potato yield loss in primary infection but a yield reduction of up to 85% has been experienced in the secondary infection [4].

To survive and avoid adverse effects under complex environmental conditions, plants have evolved a variety of surveillance mechanisms. Heat shock proteins (HSPs) were first identified as proteins induced by elevated temperatures in both prokaryotes and eukaryotes. However, HSPs also contribute to responses to other environmental stresses, including drought, cold, and salinity, and offer protection against pathogens [5,6]. Plant HSPs have been classified into six families, including HSP40, HSP60, HSP70, HSP90, HSP100, and small HSP (sHSP), according to the approximate molecular weight [7,8,9,10]. Among them, the members of HSP40, HSP70, and HSP90 families are the most abundant, and their structures and biological functions have attracted increasing attention. Previous studies have confirmed a high degree of conservation in the structures of HSPs in these three families. HSP40, also known as DnaJ, is characterized by the presence of a J-domain, generally followed by a proximal glycine- and phenylalanine-rich domain (G/F domain), a distal cysteine-rich zinc-finger domain (CXXCXGXG), and a non-conserved C-terminal domain [11,12]. HSP70 typically consists of a 44 kDa N-terminal ATPase domain (NBD), 18 kDa substrate-binding domain (SBD), and 10 kDa variable C-terminal “lid” [13]. HSP90 also contains 3 functional domains: the 12 kDa N-terminal domain containing the hydrolysis and ATP binding sites, the 35 kDa middle domain containing an amphipathic loop, and the 25 kDa C-terminal domain with a dimerization region that binds to the substrate [14,15].

Although HSPs are ubiquitous constitutive proteins induced by heat and other abiotic stresses, increasing evidence indicates that HSP members are widely involved in plant-virus interactions. For example, PVY infection induced the HSP signaling pathway and modulated the HSP response triggered by heat stress in susceptible hosts [3,16], and in turn the application of heat shock before or after inoculation could accelerate PVY propagation in tobacco plants [17]. Moreover, a DnaJ protein from *Nicotiana tabacum* (NtMPIP1) was demonstrated to interact with the movement protein of *tobacco mosaic virus* (TMV), and the silencing of this gene remarkably hindered the movement of TMV [18]. Similarly, a DnaJ protein from *N. benthamiana* (NbDnaJ) could interact with *Potato virus X* (PVX) stem-loop 1 RNA and capsid protein and played negative roles in PVX replication and movement [19]. HSP70 and its co-chaperone CPIP in *N. benthamiana* promote *Potato virus A* (PVA) infection by regulating viral capsid protein (CP) functions [20], and further investigation revealed that they are essential for PVA replication and CP accumulation [21]. In *Red clover necrotic mosaic virus* (RCNMV), host HSP70 and HSP90 interacting with p27, a virus-encoded component of the 480 kDa replication complex on the ER membrane, are required for viral RNA replication [22]. Furthermore, *N. benthamiana* HSP90 interacts with Tm-2^2^, encoding a coiled coil—nucleotide binding site—leucine-rich repeat type resistance protein, and the silencing of HSP90 reduced the steady-state levels of Tm-2^2^ protein and Tm-2^2^-mediated resistance to TMV [23].

The above findings indicate that HSP members should be conservative components recruited by viruses to facilitate their infection cycle. However, their global expression profile and the core members recruited by viruses are largely unknown in potato. Moreover, the potato *DnaJ* gene family has not been identified yet, although the *HSP70* and *HSP90* gene families have been characterized in this crop previously [24,25]. In this study, we firstly screened all members of the *DnaJ* family in the potato genome through bioinformatic methods. Subsequently, the gene expression patterns of the members in the *DnaJ*, *HSP70*, and *HSP90* gene families were investigated under PVY invasion using RNA-seq data and the qRT-PCR method, respectively. At last, the core HSP members involved in potato-PVY interaction were identified by VIGS in *N. benthamiana*. Our findings provide an overview of the core members in three major HSP families that possibly participate in PVY infection, which helps to explore the mechanism of potato-PVY compatible interaction, and benefits breeding work aiming to produce new cultivars with the capacity to grow healthily under PVY infection.

## 2. Materials and Methods

### 2.1. Identification of the DnaJ Genes in Potato

Potato genomic sequences obtained from Potato Genome Sequencing Consortium (PGSC, http://solanaceae.plantbiology.msu.edu/pgsc_download.shtml, accessed on 2 January 2022) were used to construct the local database using BioEdit7.0 software. The Hidden Markov Model (HMM) profile of the J-domain (PF00226) was acquired from Pfam (http://pfam.xfam.org, accessed on 12 January 2022) and set up as queries to blast against putative potato *DnaJ* genes with *e*-value < 10^−5^. Additionally, the keywords “HSP40”, “J-protein”, and “DnaJ” were applied to blast against the PGSC database. After redundant sequences were removed, putative DnaJ sequences were analyzed by Pfam and Smart (http://smart.embl-heidelberg.de, accessed on 15 January 2022) to verify the conserved domains. All the non-redundant genes with high confidence were named *S. tuberosum DnaJ* (*StDnaJ*) according to their location on the chromosome.

### 2.2. Sequence Analysis and Classification of Potato DnaJ Genes

Newly identified StDnaJ amino acid sequences were submitted to EXPASY PROTOPARAM (https://web.expasy.org/protparam/, accessed on 16 January 2022) for computation of the isoelectric points (PI), molecular weights (MW), and amino acid numbers. The intron numbers and chromosome locations of the *StDnaJs* were determined using the PGSC database. The potato DnaJ proteins were classified based on the composition of the domains predicted by Pfam and Smart.

### 2.3. Phylogenetic Analysis of Potato DnaJ Genes

All protein sequences of the potato *DnaJ* genes were downloaded from the PGSC database and then imported into the ClustalX program to perform multiple sequence alignments with default parameters. MEGA11.0 software was used for constructing the unrooted phylogenetic tree using the neighbor-joining method with 1000 bootstrap replicates.

### 2.4. Chromosomal Distribution and Gene Duplication Events

The mapping of *StDnaJ* genes’ chromosomal positions and relative distances was performed by MapChart2.32. Tandem duplication and segmental duplication events were analyzed. In total, 3 criteria were applied for tandem duplication: (1) at least 2 *StDnaJs* were identified within a size range of 100 kb; (2) the alignments of these *DnaJs* were observed to have a high coverage rate of the longer gene (≥70%); and (3) the identity of the aligned region was also greater than 70% [26]. The determination of the segmentally duplicated genes was conducted based on the Plant Genome Duplication Database (PGDD, http://chibba.agtec.uga.edu/duplication/index/locus, accessed on 20 January 2022).

### 2.5. Plant Materials

The potato cultivar ‘Eshu 3’ and ‘Exploits’ together with *N. benthamiana* plants preserved in our laboratory were used in this experiment. ‘Eshu 3’ is a cultivar from China that is susceptible to PVY^O^ while ‘Exploits’ from Canada possesses a temperature-dependent hypersensitive resistance to PVY^O^, and is susceptible to PVY^O^ at high temperatures (30 °C) [27]. The potato tissue culture plantlets, which tested free of PVY, were transplanted into 12-cm pots with premixed soil and grown in a greenhouse at 22 °C (16 h light/8 h dark photoperiod) and a relative humidity of 70%. The one-month-old potato plants were subjected to PVY inoculation. The two-week-old *N. benthamiana* plants, which is the time when the cotyledons and the first 2–4 true leaves emerged, were used for virus-induced gene silencing [28].

### 2.6. PVY Inoculation

The PVY^O^-FL strain was used for inoculation in this study [29]. The one-month-old potato plants (including ‘Eshu 3’ and ‘Exploits’) were mechanically inoculated with the phosphate buffer containing PVY^O^-FL isolate according to the method described previously [30]. The plants treated with blank buffer served as controls. The inoculated potato plants were grown in a greenhouse (for ‘Eshu 3’) or transferred to a growth chamber with the temperature increased to 30 °C (for ‘Exploits’). The upper systematic leaves were collected at 15 dpi (days post-inoculation) as samples for RNA isolation.

For gene-silenced *N. benthamiana* plants, PVY^O^-FL tagged with the green fluorescent protein (GFP) was cloned into the vector pCB301-2μ-HDV (kindly provided by Zhenghe Li, Zhejiang University) to construct the recombinant PVY^O^-full-length cDNA clone (pCB301-2μ:PVY^O^-FL-GFP, not published). The recombinant binary plasmids were transformed into the *Agrobacterium. tumefaciens* strain GV3101 by electroporation. The agrobacteria were grown overnight at 28 °C with shaking and then resuspended with the MMA buffer (10 mM morpholineethanesulfonic acid, 10 mM MgCl_2_, 200 μm acetosyringone, pH 5.6) to OD_600_ = 0.5. In total, 3 middle-upper leaves of each plant were selected and injected with 0.5 mL of agrobacterium solution. The accumulation of PVY in the upper uninoculated systematic leaves was investigated at 8 dpi.

### 2.7. RNA-Seq Data Analysis

To fully understand the expression patterns of the 3 potato HSP families under PVY infection, the upper leaf samples from 2 potato varieties (‘Eshu 3’ and ‘Exploits’) during PVY infection were harvested with 3 biological replicates after the plantlets were treated for 15 days. Then, the expression patterns of the three heat shock protein families were investigated from RNA-seq data, which has been not published yet (Table S1). TPM (transcripts per kilobase of exon model per million mapped reads) data were normalized to the control and then log2 transformed. The rarely expressed genes (TPM ≤ 5.0) were removed. The heat maps were generated by TBtools software [31].

### 2.8. Virus-Induced Gene Silencing

Virus-induced gene silencing (VIGS) has been applied routinely in *N. benthamiana* as a way to verify the functions of candidate genes and to discover new genes required for diverse pathways, especially disease resistance signaling [32]. To construct new vectors for VIGS, the fragments of 7 target genes were amplified using the specific primers (Table S1), verified by the VIGS tool in SGN (https://vigs.solgenomics.net/, accessed on 3 February 2022), and cloned into the TRV (tobacco rattle virus) RNA2 vector. The empty vector TRV RNA2 was set as the control. The pTRV1, pTRV2, and 7 new pTRV2 vectors were transformed into the *A. tumefaciens* strain GV3101 by electroporation, respectively. The mixture of *Agrobacterium* cultures containing pTRV1 and pTRV2 (1:1, *v*/*v*) and pTRV1 and pTRV2-T-target genes (1:1, *v*/*v*) at OD_600_ = 0.5 was incubated for 3 h in the darkness at room temperature before inoculation. The gene silencing efficiency was tested by qRT-PCR (the primers are shown in Table S1). Three independent replicates were performed.

### 2.9. RNA Isolation, qRT-PCR, and ELISA Analysis

The total RNA was isolated by the Plant Total RNA Kit (Zoman, Beijing, China) following the manufacturer’s protocol. The cDNA was synthesized by HiScript II Reverse Transcriptase (Vazyme, Nanjing, China). The gene-specific primers were designed by Primer Premier 5.0 (Table S1). The *Stef1α* (AB061263) and *Ntactin* (XM_016658880) genes were set up as internal controls. Quantitative real-time polymerase chain reaction (qRT-PCR) was carried out in an optical 96-well plate with a CFX Connect TM Real-Time System q-PCR machine (Bio-rad, California, CA, USA). The reaction volume consisted of 5 μL of EVAGreen Express 2xqPCR MasterMix (ABM, Vancouver, BC, Canada), 1 μL of cDNA, 0.3 μL of each primer (10 μM), and 3.4 μL of ddH_2_O. The PCR program was set as follows: 95 °C for 3 min, 40 cycles at 95 °C for 10 s, 55 °C for 15 s, and 72 °C for 20 s. Three technical replications were performed for each sample. The obtained data were dealt with the 2^−^^Δ^^Δ^^ct^ method [33]. The enzyme-linked immunosorbent assay (ELISA) was carried out according to the method previously described [27].

## 3. Results

### 3.1. Identification, Classification, and Phylogenetic Analysis of Potato DnaJ Genes

To achieve an overview of the potato *DnaJ* genes, HMM and keywords searches were performed using the PGSC database. After the removal of redundant sequences, the maintained sequences were subjected to analysis by Pfam and SMART to verify the J-domain and other conserved domains. Finally, 89 sequences were assigned as *S. tuberosum* *DnaJ* (*StDnaJ*) genes and named *StDnaJ01* to *StDnaJ89* according to their location on the chromosome. The biological information of each *StDnaJ* is shown in Table S2, including the gene name, gene ID, chromosomal location, amino acid number, intron number, molecular weight (MW), domain number, and isoelectric point (pI).

Based on the presence/absence of 3 characterized domains identified in StDnaJ proteins, the J-domain, zinc-finger domain (CXXCXGXG), and C-terminal domain, the StDnaJ proteins were divided into 4 groups (I, II, III, and IV) with 9, 8, 67, and 5 members, respectively (Table S2). Group I StDnaJ proteins comprise all three domains; Group II holds the J-domain and C-terminal domain but not the zinc-finger domain; Group III only has the J-domain; and Group IV contains a J-domain without the HPD motif, which have been characterized as DnaJ-like proteins [34].

**Figure 1:**
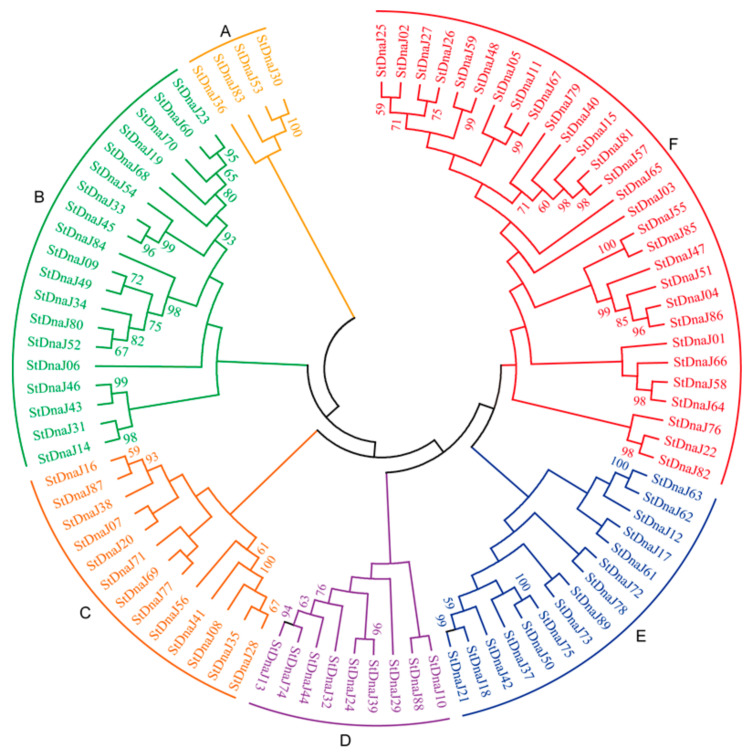
Phylogenetic relationship of the 89 *StDnaJ* genes in potato. These genes were clustered into six subfamilies (A, B, C, D, E, and F). The unrooted tree was constructed by the neighbor-joining method with 1000 bootstrap replicates through MEGA 11.0 software.

According to sequence homology, the 89 *StDnaJ* genes were segmented into 6 subfamilies (sub-family A, B, C, D, E, and F) (Figure 1). Sub-family A was the smallest group (containing only 4 members), followed by sub-family D (9 members), sub-family C (13 members), sub-family E (15 members), and sub-family B (19 members). Sub-family F was the largest group, with up to 29 *StDnaJ* gene members. This result is not consistent with the classification according to the domains, indicating the evolutionary diversity of the *DnaJ* gene family in potato.

### 3.2. Chromosomal Locations and Gene Duplication Events of the StDnaJ Family

**Figure 2:**
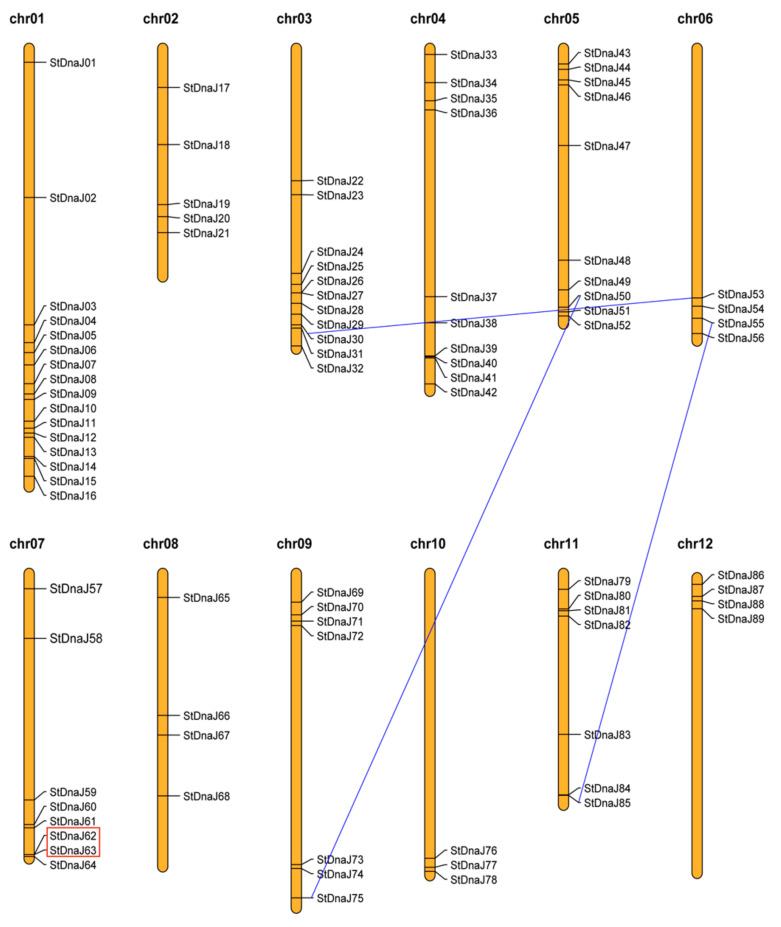
Chromosomal locations and gene duplication events of the 89 *StDnaJ* genes. The tandem duplicated genes are labeled with a red rectangle and the segmental genes are denoted by blue lines. The map was generated by MapChart2.32.

The 89 *StDnaJs* were randomly mapped on 12 chromosomes, and most of them were identified on the proximate or distal ends of the chromosomes. Among them, in the order from more to less, 16 *StDnaJs* were located on chromosome 1; 11 on chromosomes 3; 10 on chromosome 4 and 5; 8 on chromosome 7; 7 on chromosomes 9 and 11; 5 on chromosomes 2; 4 on chromosomes 6, 8, and 12; and 3 on chromosome 10 (Figure 2).

Based on the defined criteria, 2 tandem duplicated genes (*StDnaJ62* and *StDnaJ63*) were found on chromosome 7. This pair of *StDnaJ* genes was mapped proximally in distance and separated by less than one gene. Six segmentally duplicated genes were found. *StDnaJ30* on chromosome 3 exhibited synteny with *StDnaJ53* on chromosome 6. Similar duplication events were also discovered for *StDnaJ50* on chromosome 5 and *StDnaJ75* on chromosome 9, and *StDnaJ55* on chromosome 6 and *StDnaJ85* on chromosome 11 (Figure 2). These results suggest a possible evolutionary mechanism of potato *DnaJ* genes by dominant segmental duplications.

### 3.3. Expression Profiles of StDnaJ, StHSP70, and StHSP90 Families under PVY Infection

**Figure 3:**
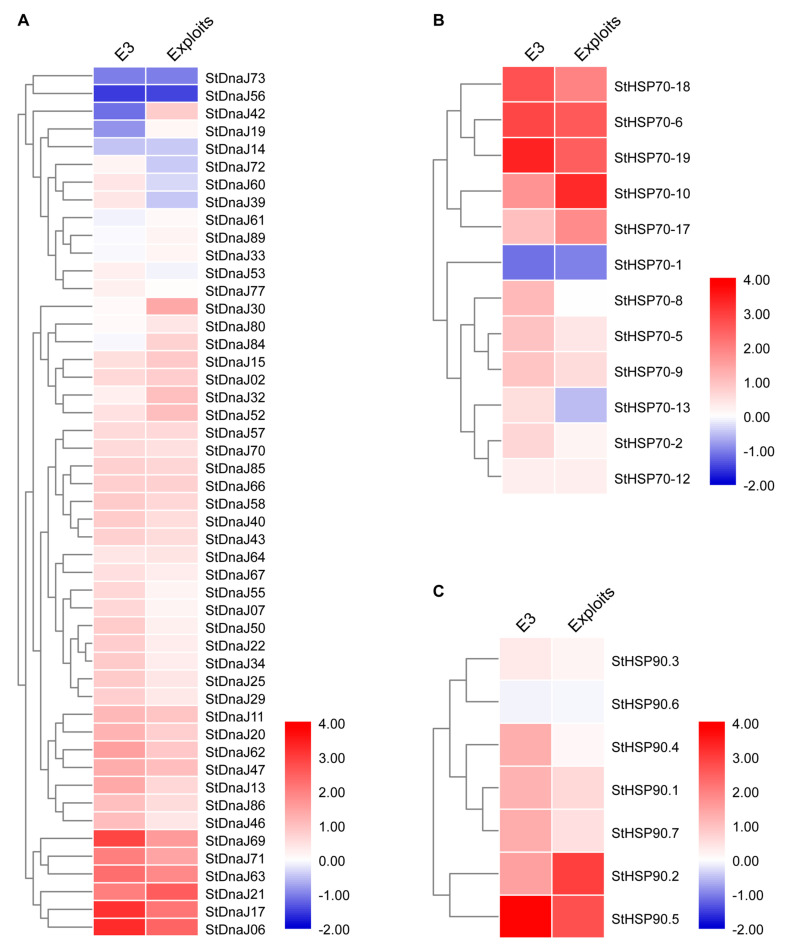
Expression profiles of the members in three major HSP families under PVY infection in two potato cultivars based on RNA-seq data. (**A**) The expression profile of the *StDnaJ* family; (**B**) the expression profile of the *StHSP70* family; (**C**) the expression profile of the *StHSP90* family. Only HSP genes with a minimum TPM (>5) were included in the analysis, and the TPM data were normalized to the control and then log2 transformed. The heat maps were generated by TBtools software. Red, white, and blue elements indicate upregulated, regular, and downregulated, respectively.

The potato cultivar ‘Eshu 3’ is susceptible to PVY^O^ while ‘Exploits’ is only susceptible at the high temperature (30 °C) [27,35]. To verify the expression profiles of the members in three HSP families (*StDnaJ*, *StHSP70*, and *StHSP90*) during PVY^O^ infection, the upper systematic leaves were collected from ‘Eshu 3’ and ‘Exploits’ in compatible interactions with PVY at 15 dpi and used for RNA-seq analysis (Figure 3, Table S3).

A total of 89 *StDnaJs*, 20 *StHSP90s*, and 7 *StHSP90s* were identified in this study and previous studies [24,25]. Based on the RNA-seq data, 49 *StDnaJs*, 12 *StHSP90s*, and 7 *StHSP90s* exhibiting the minimum expression level (TPM > 5) were included in the further analysis (Table S3). The 78 members in 3 major HSP gene families presented different expression profiles in 2 potato cultivars under PVY infection (Figure 3, Table S3). In the *StDnaJ* family, most members were upregulated, especially *StDnaJ06*, *StDnaJ17*, *StDnaJ21*, *StDnaJ63*, *StDnaJ69*, and *StDnaJ71*, which were remarkably induced (>2-fold) in 2 potato cultivars. A few members were downregulated in one or two potato cultivars, for example, *StDnaJ19* and *StDnaJ61* were only downregulated in ‘Eshu 3’ while *StDnaJ56* and *StDnaJ72* were inhibited (>2-fold) in both cultivars (Table S3 and Figure 3A). In the *StHSP70* family, *StHSP70-1* was downregulated in two cultivars, and *StHSP70-8* and *StHSP70-13* were inhibited only in ‘Exploits’. The remaining members exhibited upregulated expression levels, and 5 of them (*StHSP70-6*, *StHSP70-10*, *StHSP70-17*, *StHSP70-18*, and *StHSP70-19*) were significantly upregulated (>2-fold) in both potato cultivars (Table S3 and Figure 3b). In the *StHSP90* family, almost all members were upregulated, except for *StHSP90.6*, which was slightly downregulated in two cultivars. Among them, *StHSP90.2* and *StHSP90.5* were significantly upregulated (>2-fold) in both cultivars (Table S3 and Figure 3C).

**Figure 4:**
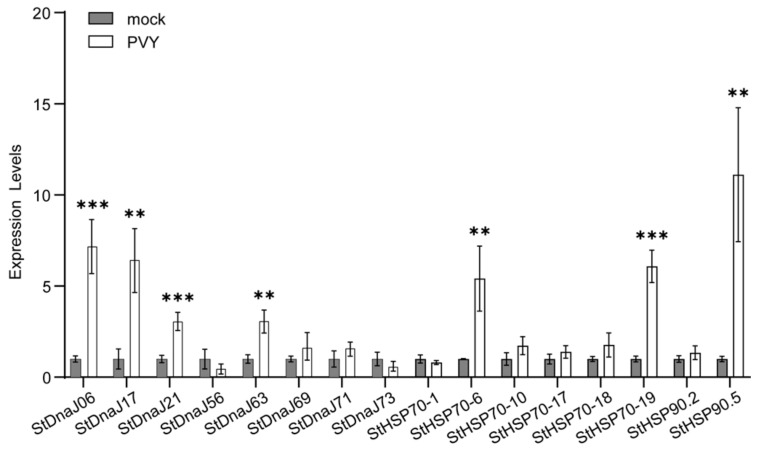
Transcript levels of the 16 candidate genes under PVY infection in ‘Eshu 3’ at 15 dpi using qRT-PCR. The data were dealt with the 2^−^^Δ^^Δ^^ct^ method and then normalized to the control (mock), which was set as 1. Statistically significant differences (*p* < 0.01 and < 0.001 (Student’s *t*-test)) are denoted by 2 and 3 asterisks, respectively. The bars represent the standard deviation (±SD) calculated for three biological replicates.

Based on the RNA-seq data, a total of 16 candidate genes (including *StDnaJ06*, *StDnaJ17*, *StDnaJ21*, *StDnaJ56*, *StDnaJ63*, *StDnaJ69*, *StDnaJ71*, *StDnaJ73*, *StHSP70-1*, *StHSP70-6*, *StHSP70-10*, *StHSP70-17*, *StHSP70-18*, *StHSP70-19*, *StHSP90.2*, and *StHSP90.5*) were remarkably upregulated (>2-fold) or downregulated (>2-fold) under PVY infection. Subsequently, these genes were selected and their expression levels in response to PVY infection further confirmed in ‘Eshu 3’ using qRT-PCR (Figure 4). The results indicate that 7 HSP genes (*StDnaJ06*, *StDnaJ17*, *StDnaJ21*, *StDnaJ63*, *StHSP70-6*, *StHSP70-19*, and *StHSP90.5*) were significantly upregulated compared to the control (mocked). Therefore, we assumed that these seven genes possibly played a role in the potato-PVY interaction process.

### 3.4. Virus-Induced Gene Silencing (VIGS) Assays

To identify whether the 7 significantly upregulated genes (*StDnaJ06*, *StDnaJ17*, *StDnaJ21*, *StDnaJ63*, *StHSP70-6*, *StHSP70-19,* and *StHSP90.5*) were associated with the accumulation of PVY, we performed VIGS assays by silencing their homologous genes in the model plant *N. benthamiana*, a method frequently used to verify the functions of candidate genes in *Solanaceae*, due to its difficulty in silencing genes by VIGS in potato crop. The homologous genes in *N. benthamiana* were identified based on the highest sequence similarity to the corresponding potato genes (Table S4). Fragments of 200-300 bp specific to the homologous genes in *N. benthamiana* were used to generate the TRV2 VIGS vector.

**Figure 5:**
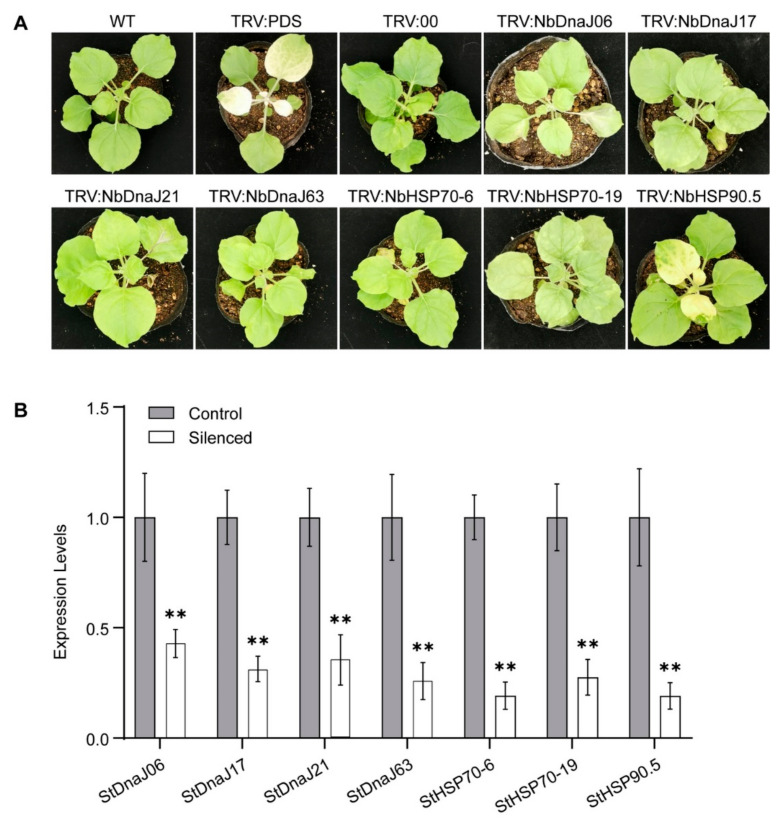
Silencing of the 7 target HSP genes in *N. benthamiana* plants. (**A**) The phenotypes of the gene-silenced *N. benthamiana* plants at 10 dpi; (**B**) Silencing efficiency of the 7 target genes. The transcript levels were tested by qRT-PCR. The gene expression data were dealt with the 2^−^^Δ^^Δ^^ct^ method and then normalized to the control (TRV: 00), which was set as 1. The bars represent the standard deviation (±SD) calculated for three biological replicates and ** denotes significance difference at *p* < 0.01 by Student’s *t*-test.

The silencing of the *phytoene desaturase* (*PDS*) gene led to plants being bleached at 10 days (Figure 5A), demonstrating the silence system was effective. In comparison with the control plants (TRV: 00), the *NbHSP90.5*-silenced plants showed obvious chlorosis phenotypes in the upper new developed leaves, indicating the possible function of this gene in chloroplast biogenesis as previously reported [36], while the silencing plants of the remaining six candidate HSP genes did not exhibit any visible difference in plant growth and development (Figure 5A). Subsequent qRT-PCR assays showed that the transcript levels of the 7 target genes were reduced by at least 50% compared to those of the control plants (Figure 5A).

**Figure 6:**
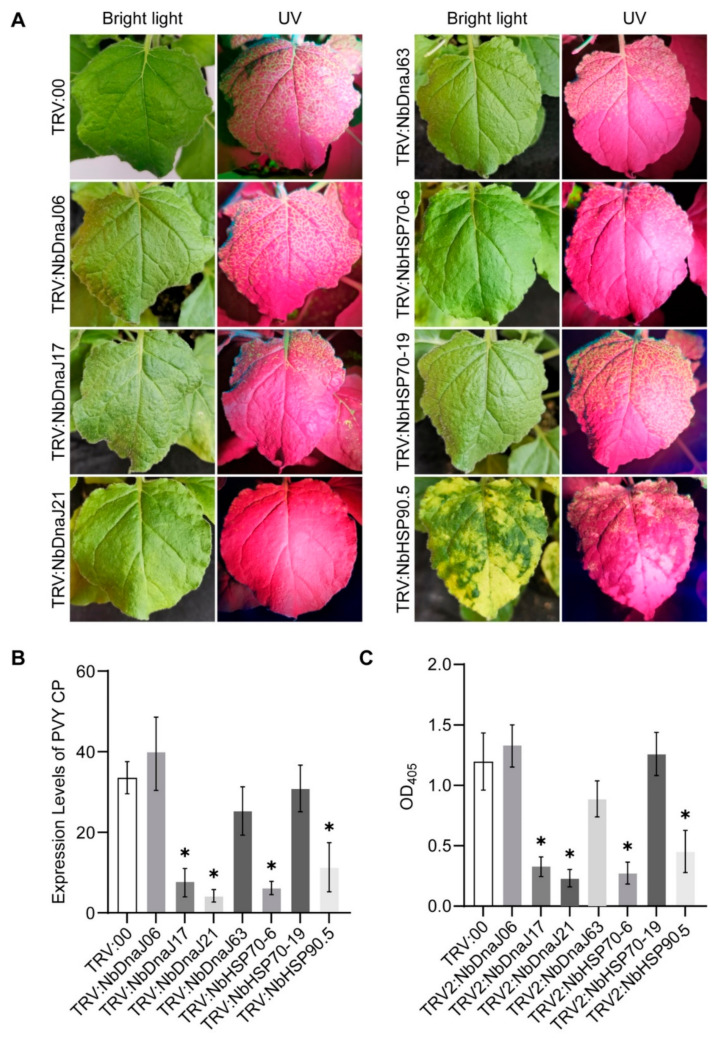
Effect of silencing the 7 target genes on PVY infection in VIGS assays. (**A**) Infection of PVY in the upper non-inoculated leaves of gene-silenced plants compared to the control (TRV:00); (**B**) Transcript levels of PVY CP by qRT-PCR; (**C**) Protein expression levels of PVY CP by ELISA. The upper leaves were collected as samples at 8 dpi. The leaf samples from a single plant were divided equally into two parts, half for qRT-PCR and the other half for ELISA. Statistically significant differences (*p* < 0.05 (Student’s *t*-test)) are denoted by an asterisk, and the bars represent the standard deviation (±SD) calculated for three biological replicates.

To test the possible functions of the above target genes in the virus infection, the gene-silenced and control tobacco plants were further infiltrated with the agrobacterium solution containing the infectious cDNA clone of PVY^O^-FL-GFP. The accumulation of PVY reflected in the fluorescence areas in plants’ upper leaves was monitored. At eight days after PVY inoculation, the area and concentration of the green fluorescence were remarkably reduced in *NbDnaJ17*-silenced (TRV: NbDnaJ17), *NbDnaJ21*-silenced (TRV: NbDnaJ21), *NbHSP70-6*-silenced (TRV: NbHSP70-6), and *NbHSP90.5*-silenced (TRV: NbHSP90.5) plants compared with the control (TRV: 00) (Figure 6A), although the green fluorescence emerged in the upper leaves of all treated plants. Meanwhile, the transcript levels of PVY CP checked by qRT-PCR were significantly lower in the silencing plants of the above 4 genes than those in the control plants at 8 dpi (Figure 6B), and the protein levels of PVY CP determined by ELISA exhibited a consistent result with those of qRT-PCR at the same time (Figure 6C). These results suggest that *DnaJ17*, *DnaJ21*, *HSP70-6*, and *HSP90.5* possibly functioned as susceptibility factors, contributing to the virus accumulation in host-PVY interaction.

## 4. Discussion

It is increasingly imperative to identify and characterize gene families in the potato genome owing to its importance for comprehending the roles of plants in response to environmental stresses. The *DnaJ* gene family has been identified in many species from bacteria to human, although the number of *DnaJs* varies in different species. However, this family had not previously been studied in potato. Therefore, a genome-wide analysis of the *DnaJ*/*HSP40* gene family was performed in potato in this study, which characterized the protein structure, classification, chromosome location, and gene duplication events of a total of 89 *DnaJ* genes in potato (Table S2). As the previous studies reported, only 6 *DnaJ* genes were identified in *Escherichia coli* [37], and 25 *DnaJ* genes were found in *Saccharomyces cerevisiae* [34], 27 *DnaJ* genes were characterized in *Bombyx mori* [38], and 41 *DnaJ* genes were described in *Homo sapiens* [39]. However, in plant species, the number of potato *DnaJ* genes was close to that found in *Capsicum annuum* and less than *Arabidopsis thaliana*, and they possessed 76 and 120 *DnaJ* genes, respectively [40,41]. All these studies showed that plants, as sessile organisms, have a larger number of *DnaJ* genes than other species, which could be beneficial for them to be challenged by complex environments.

Besides the difference in the total number, the classification of *DnaJ* genes based on the protein domain composition is also different among species. *E*. *coli* and *B. mori* contain three groups of *DnaJ* genes (I to III) [37,38]. *S.* *cerevisiae*, *H*. *sapiens*, and *A. thaliana* possess four groups of *DnaJ* genes (I to IV) [34,39,40]. According to our results, the 89 potato *DnaJ* genes were also classified into 4 groups (I to IV) (Table S2). However, the *DnaJ* genes in *C. annuum* were categorized into five groups (I to V) [41]. The results demonstrate that species tend to evolve into different groups of *DnaJ* genes to perform various functions. In addition, group III *DnaJ* genes containing only a J-domain are the most abundant in all species, indicating that they may play more complex roles or participate in multi-biological processes.

It was reported that the gene family expansions, and genome evolutionary mechanisms, primarily depended on gene duplication events [42], which mainly consists of tandem duplication and segmental duplication [43]. In this study, the 89 *StDnaJ* genes were mapped randomly across 12 chromosomes, with most of them on the terminal regions of chromosomes. Despite the fact that the potato genome size was nearly 7-fold larger than that of *A. thaliana*, there were fewer *DnaJ* genes found in potato (89 genes) than in *A. thaliana* (120 genes) [40]. The possible explanation is the difference in whole-genome duplication incidences between potato and *A. thaliana*. A total of four pairs of *StDnaJ* genes were involved in gene duplication events, including one tandem duplication (*StDnaJ62* and *StDnaJ63)* and three segmental duplication events (*StDnaJ30*/*StDnaJ53*, *StDnaJ55*/*StDnaJ85*, and *StDnaJ50*/*StDnaJ75*) (Figure 2). Therefore, we suggested that the tandem, and segmental duplications, play an indispensable role in the expansion of *StDnaJ* genes in potato, which may be dominated by the latter, although the tandem duplicate is likely important for plants to adapt evolution under rapidly changing environments [44]. Our result is similar to the observation in the pepper in which one tandem duplication event and two segmental duplication events were detected in the *DnaJ* gene family, respectively [41]. Moreover, the tandem duplications in *HSP90* gene families were obviously less than segmental duplications in 15 out of 25 plant species, including potato, and the tandem duplications did not exist in most of them (13/15) [25]. Meanwhile, in the potato *HSP70* gene family, three tandem duplication events and two segmental duplication events were found [24]. The above-mentioned findings indicate that HSP gene families may display a diverse expansion model.

Potato (*S. tuberosum* L.), belonging to *Solanaceae*, is a well-known “friendly” host for PVY. This suggests that a series of host factors (susceptibility genes) in potato can facilitate the infection of this virus, which are largely unknown. Increasing studies have reported that members of the plant *DnaJ*, *HSP70*, and *HSP90* gene families play important roles in the host-virus interaction process [19,21,23]. However, their global expression profiles and core members recruited by viruses are not clear in potato. In this study, the global expression profiles of the three HSP genes families were investigated based on the RNA-seq data (Figure 3). Out of them, 16 genes (*StDnaJ06*, *StDnaJ17*, *StDnaJ21*, *StDnaJ56*, *StDnaJ63*, *StDnaJ69*, *StDnaJ71*, *StDnaJ73*, *StHSP70-1*, *StHSP70-6*, *StHSP70-10*, *StHSP70-17*, *StHSP70-18*, *StHSP70-19*, *StHSP90.2*, and *StHSP90.5*) were differently expressed genes (*DEGs*) in 2 potato cultivars during PVY infection (Figure 3 and Figure 4). Interestingly, six out of eight *DEGs* in the *StDnaJ* family were group III *DnaJ* genes, and the remaining two (*StDnaJ06, StDnaJ56*) belonged to group II and IV, respectively (Table S2), which indicates the J-domain may play an important role in the interaction of DnaJ HSPs and PVY. Among them, *StDnaJ69* shares 92% and 96% identity with *N. benthamiana P58^IPK^* (*NbP58^IPK^*) and tomato *P58^IPK^* (*LeP58^IPK^*) at the nucleic acid level, respectively. P58^IPK^ contains nine tetratricopeptide repeats (TPRs) arranged in tandem at the N terminus and a J-domain at the C terminus, which is conservative in plants and animals [45,46]. In tobacco, NbP58^IPK^ can interact with the TMV-P50 and TEV-helicase and functions as a susceptibility gene required for virus virulence [45]. In animals, P58^IPK^ is recruited by the influenza virus and Hantaviruses to combat the protein kinase R (PKR)-mediated innate antiviral response [46]. Another StHSP70-17 is highly homologous to the tomato Hsc70.3, which was proved to interact with the capsid protein (CP) of *Pepino mosaic virus* (PepMV) [47], and recently, its homologue *NbHSP70C* was reported to be inhibited by a natural antiviral agent (quercetin) in tobacco to disturb the replication of TMV [48].

Further qRT-PCR analysis on the above differently expressed genes revealed that seven HSP genes (*StDnaJ06*, *StDnaJ17*, *StDnaJ21*, *StDnaJ63*, *StHSP70-6*, *StHSP70-19,* and *StHSP90.5*) were significantly induced in ‘Eshu 3’ under PVY infection (Figure 4), and their homologous genes in *N. benthamiana* were subsequently investigated by VIGS assays. The silencing of four genes (*DnaJ17*, *DnaJ21*, *HSP70-6*, and *HSP90.5*) dramatically reduced the accumulation of PVY in *N. benthamiana* plants (Figure 6), suggesting their possible roles in the infection of PVY. Intriguingly, *StHSP90.5* is highly homologous to Arabidopsis *HSP90.5* and tobacco *Hsp90C*, which are chloroplast-localized HSP90 family molecular chaperones [49]. In Arabidopsis, HSP90.5 has been proposed to play essential roles in chloroplast biogenesis, protein folding in the chloroplast, and the transport of proteins into chloroplasts [49,50,51,52]. The knockdown of chloroplast HSP90 leads to variegated or albino phenotypes in both Arabidopsis and tobacco [36,49], although the knockout of the gene is embryonically lethal in Arabidopsis [51,52]. Similar chlorotic phenomena were observed in *HSP90.5*-silenced tobacco plants in our VIGS assay (Figure 5 and Figure 6). In the molecular events behind the phenotype resulting from the suppression of chloroplast HSP90, the chloroplast- and photosynthesis-related genes were downregulated as expected [36,53]. Additionally, it is noteworthy that the defense-related genes, such as immunity/pathogenesis-related genes and genes involved in the response to oxidative stress and the cell death pathway, were upregulated [36,53]. Moreover, cell death and H_2_O_2_ production were detected in the leaves developing chlorosis [53]. Together with the fact that chlorosis is a common symptom induced by a virus or viroid, we propose that chloroplast HSP90 may be a core susceptibility factor recruited by these viral pathogens for counter-defense and inducing disease symptoms. Indeed, the chloroplast *HSP90* in peach has been reported to be targeted for cleavage by two small RNAs derived from *Peach latent mosaic viroid* (PLMVd), a chloroplast-replicating viroid inducing clear bleaching-type chlorosis [54]. Unfortunately, the experiments did not test the impact of chloroplast *HSP90* knockdown on the resistance or susceptibility to the viroid in the transgenic plants. Our results of the VIGS assays indicated that *NbHSP90.5*, together with *NbDnaJ17*, *NbDnaJ21*, and *NbHSP70-6*, might be recruited as susceptibility factors to facilitate PVY infection, which partially supported our above hypothesis. Although *NbDnaJ06*, *NbDnaJ63*, and *NbHSP70-19* were markedly induced by PVY infection, the silencing of these genes did not significantly suppress or promote PVY accumulation in the VIGS assays. Our data indicate that these genes may not be indispensable for PVY accumulation. Nevertheless, this result cannot exclude the possibility of these genes being involved in PVY infection since knockdown of these genes by RNA interference is incomplete compared with knockout by the CRISPR/Cas9 systems. Clearly, the functions and underlying mechanisms of these *HSPs* involved in the host-virus interactions need to be clarified by further study.

## 5. Conclusions

In summary, a total of 89 *StDnaJ* genes were identified in the potato genome in this study. A series of analyses of this gene family, including their classification, phylogenetic relationships, chromosomal locations, and gene duplication events, were carried out using bioinformatics methods. Moreover, the RNA-seq data and qRT-PCR analysis demonstrated that *StDnaJ06*, *StDnaJ17*, *StDnaJ21*, *StDnaJ63*, *StHSP70-6*, *StHSP70-19*, and *StHSP90.5* were significantly upregulated during the PVY infection process, implying their potential roles in the potato-PVY compatible interaction process. Further, the knockdown of the homologous genes of *StDnaJ17*, *StDnaJ21*, *StHSP70-6*, and *StHSP90.5* in *N. benthamiana* by VIGS dramatically reduced the accumulation of PVY, which indicates that the four genes may function as susceptibility factors in the PVY infection process. Our work provides candidate genes that possibly participate in the plant-virus interactions in the three major HSP families, which helps to understand the mechanism of potato-PVY compatible interaction, and benefits breeding work aiming to produce new cultivars with the capacity to grow healthily under PVY infection.

## Data Availability

The datasets supporting the conclusions of this article are included within the article.

## Supplementary Materials

The following supporting information can be downloaded at: https://www.mdpi.com/article/10.3390/genes13040566/s1. Table S1: The specific primers for qRT-PCR and VIGS vectors; Table S2: The bioinformation of *DnaJ* genes identified in potato; Table S3: The RNA-seq data used for the analysis of expression profiles of the three potato HSP families. Table S4: The highest homologous genes of the seven potato HSP target genes in *N. benthamiana*.
